# A multiplex PCR assay for the identification of five species of the *Anopheles barbirostris* complex in Thailand

**DOI:** 10.1186/s13071-019-3494-8

**Published:** 2019-05-14

**Authors:** Laura Brosseau, Chanya Udom, Chutipong Sukkanon, Theeraphap Chareonviriyaphap, Michael J. Bangs, Atiporn Saeung, Sylvie Manguin

**Affiliations:** 1 HydroSciences Montpellier (HSM), Institut de Recherche pour le Développement (IRD), CNRS, Université Montpellier, Montpellier, France; 2 Department of Zoology, Faculty of Science, Kasetsart University, Bangkok, 10900 Thailand; 3 Department of Entomology, Faculty of Agriculture, Kasetsart University, Bangkok, 10900 Thailand; 4 Department of Public Health and Malaria Control, International SOS, Jl. Kertajasa, Kuala Kencana, Papua, 99920 Indonesia; 5 Department of Parasitology, Faculty of Medicine, Chiang Mai University, Chiang Mai, 50200 Thailand

**Keywords:** *Anopheles*, Barbirostris Complex, ITS2, multiplex PCR, Thailand

## Abstract

**Background:**

The Barbirostris Complex comprises six formally described species that cannot be differentiated based on morphology alone. Out of these six species, two have been reported as putative malaria vectors, *An. campestris* and *An. wejchoochotei*. Five species are present in Thailand, *An. barbirostris*, *An. campestris*, *An. dissidens*, *An. saeungae* and *An. wejchoochotei*, while *An*. *vanderwulpi* occurs in Indonesia. As these species cannot be accurately differentiated by morphological characters, there is a crucial lack of information on their bionomics and role in the transmission of malaria and filariasis agents.

**Results:**

For differentiating the six species, an allele-specific amplification (AS-PCR) based on the second internal transcribed spacer (ITS2) sequence was developed. From 862 mosquitoes in the Barbirostris Complex collected in 23 provinces throughout Thailand, the AS-PCR was able to identify five species and its validation was undertaken on 185 specimens.

**Conclusions:**

This multiplex-PCR assay is potentially able to definitely identify all six species of the Barbirostris Complex and was validated on five species present in Thailand.

## Background

*Anopheles* (*Anopheles*) *barbirostris* belongs to the Barbirostris Complex within the Barbirostris Group of the Myzorhynchus Series [1]. Recently, Taai & Harbach [2] described within the Barbirostris Complex three new species, *An. dissidens*, *An. saeungae* and *An. wejchoochotei*, which accounts for six formally named species including *An. barbirostris*, *An. vanderwulpi* and *An. campestris*, the latter one being recognized as a member of this complex [2]. Four species are reported as primarily zoophilic throughout their geographic range, although they may bite humans in the absence of their usual hosts (typically bovids). The two others, *An. wejchoochotei* and *An. campestris*, are known for their greater anthropophilic behavior, especially the latter species that more readily bites humans than any other members of the Barbirostris Complex [2, 3]. *Anopheles barbirostris* (*s.l*.) is widely distributed in Thailand [4, 5] and more globally in the Asian region [2, 6–8]. It has been reported as a vector of *Plasmodium falciparum* and *Plasmodium vivax* in Sri Lanka, Bangladesh, Indonesia (Sumatra, Sulawesi, Flores), Timor Leste, as well as a secondary vector on the island of Borneo [9] and a putative malaria vector in the Aranyaprathet District, Sa Kaeo Province, southeastern Thailand [10, 11]. More specifically, *An. barbirostris* (*s.l*.), *An. campestris* and *An. wejchoochotei* (former ‘*campestris*-like’, see Table 1) have been incriminated as vectors of *P. falciparum* and *P. vivax* [2–4, 11–17]. However, the lack of reliable methods to identify the species within the complex has hampered precise evaluation of the specific role of each member in transmission of malaria and other pathogens, e.g. *Brugia timori* and *Brugia malayi* in Indonesia [12, 29].

**Table 1:** Correspondence of formally named species in the Barbirostris Complex based on six studies [23–25, 35, 36, 38] and the ITS2 length of the dominant product [2, 24], known geographical distribution, biting behavior [2, 8] and experimental infection studies [10, 17]

*Anopheles* species	Reference				ITS2 length (bp)	Confirmed distribution	Biting behavior	Experimental infections
	[35]	[25, 38]	[36]	[23]				
*An. barbirostris*	X	–	A4	Clade 1	1637	Indonesia, Thailand, Vietnam	Mainly zoophilic	Negative for Pf and Pv
*An. dissidens*	–	A1	–	Clade III	1822	Thailand	Mainly zoophilic	Low positivity for Pv (9.1%)
*An. saeungae*	–	A2	–	Clade IV	1678	Indonesia, Thailand	Mainly zoophilic	Low positivity for Pv (6.7%)
*An. vanderwulpi*	W	–	–	Clade II	1727	Indonesia	Mainly zoophilic	–
*An. wejchoochotei*	–	*An. campestris*-like^a^		Clade V	1612	Thailand	Anthropophilic	High positivity for Pv (> 60%)
*An. campestris*	–	-	–	–	1519	Malaysia, Thailand	Anthropophilic; malaria vector	High positivity for Pv

^a^*An. campestris*-like (= *An. wejchoochotei*) originally described by Harrison & Scanlon [3]

*Abbreviations*: Pf, *Plasmodium falciparum*; Pv, *Plasmodium vivax*

The aim of this study was to develop a rapid and accurate identification method to distinguish the known species of the Barbirostris Complex. The principle of allele-specific PCR (AS-PCR) methodology was selected based on species-specific differences within the sequences of the internal transcribed spacer 2 (ITS2), a ribosomal DNA gene (rDNA) widely used to differentiate cryptic species of *Anopheles*, particularly those belonging to Asian complexes and groups [18–22].

## Methods

### Primer design based on ITS2 sequences

Allele-specific primers were designed from the rDNA ITS2 sequences from previous studies [2, 23–25]. ITS2 sequences were aligned using Multalin version 5.4.1 [26] to obtain a consensus sequence for each species, which was used to determine specific primers. Primers were designed manually and using the Primer3 input (version 0.4.0) program [27]. The melting temperatures of the primers were kept similar to each other so that they could be combined readily in a single PCR set-up. The oligonucleotide primers were synthetized by Eurogentec (Belgium).

### Multiplex allele-specific PCR

To identify the six species of the Barbirostris Complex, nine primers were designed. To avoid high competition between the primers and according to melting temperatures, a double multiplex PCR was developed. The first PCR (PCR1) was designed to identify *An. barbirostris*, *An. vanderwulpi*, *An. dissidens* and *An. campestris*, while the second PCR (PCR2) focused on differentiating *An. saeungae* and *An. wejchoochotei.* Both PCR1 and PCR2 were carried out using 25 µl volumes containing 1 unit of GoTaq^®^ G2 Flexi DNA Polymerase, 1× GoTaq^®^ Flexi Buffer, 1.5 mM MgCl_2_ (enzyme, buffer and MgCl_2_ supplied by Promega Corporation, Madison, WI, USA), 200 µM dNTP, each primer at 0.15 µM and 0.5 µl of extracted DNA. The PCR conditions were carried out at 95 °C for 1 min, followed by 35 cycles at 95 °C for 30 s, 44 °C (PCR1) or 51 °C (PCR2) for 30 s and 72 °C for 1 min, with a final extension step at 72 °C for 10 min. The PCR products were subjected to electrophoresis on a 2% agarose gel stained with GelRed (Biotium Inc, Fremont, CA, USA).

### Mosquito collection, morphological identification and DNA extraction for PCR assay validation

**Fig. 1:**
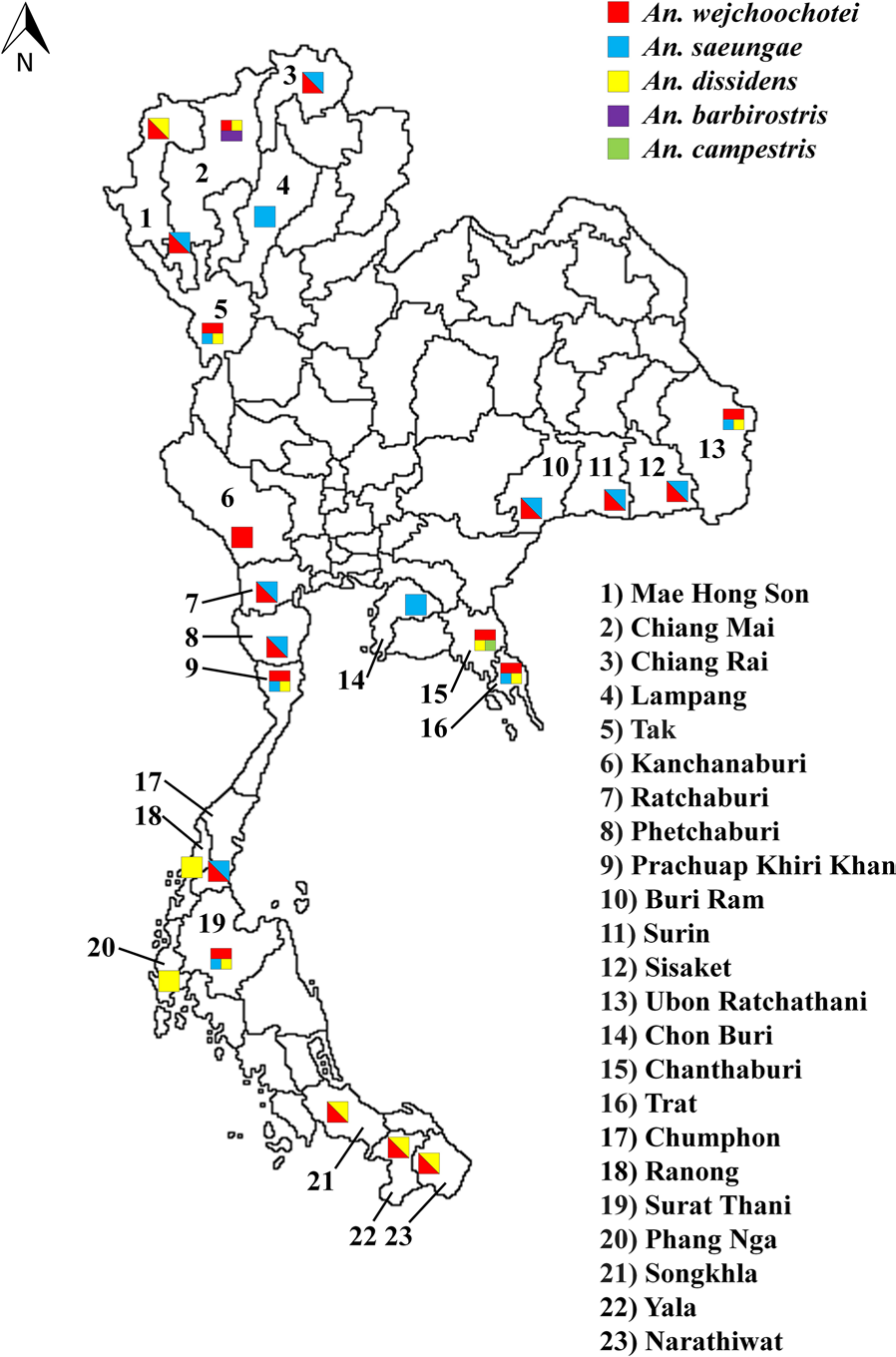
Localizations (squares) of the mosquito sampling sites according to the approximate proportion of five species in the Barbirostris Complex collected in 23 provinces of Thailand

During December 2016 and March 2017, 862 specimens of the Barbirostris Complex were collected in 23 provinces of Thailand (Fig. 1). Mosquito collections were done between 18:00 and 24:00 h by human-landing catches or cow-baited trapping (tents or landing catches) depending on the locality. Females were individually placed in 1.5 ml Eppendorf tubes and preserved by desiccation with silica gel. Morphological identification of mosquitoes was performed at Kasetsart University using standard illustrated keys allowing the separation of three taxa, *An. barbirostris*, *An. campestris*, and a third one called “unknown species” [23]. Genomic DNA was extracted from whole individual adult mosquitoes based on routine procedures [18]. Of 434 samples amplified by AS-PCR, 43 samples were subsequently sequenced for species confirmation and PCR assay validation.

### Amplification and sequencing of the ITS2 region

To confirm the results of the dual multiplex PCR, the ITS2 rDNA of 43 samples with four different profiles were amplified using universal primers ITS2A (5′-TGT GAA CTG CAG GAC ACA T-3′) and ITS2B (5′-TAT GCT TAA ATT CAG GGG GT-3′) [25, 28]. Reactions were performed in a 50 µl volume using a “ProFlex” thermal cycler (Thermo Fisher Scientific, Waltham, MA, USA). Each tube contained 1 µl of mosquito DNA from the previous extractions, each primer at 0.2 µM, 200 µM dNTP, 1.5 mM MgCl_2_, 1× GoTaq^®^ Flexi Buffer and 2 units of GoTaq^®^ G2 Flexi DNA Polymerase (enzyme, buffer and MgCl_2_ supplied by Promega Corporation, Madison, WI, USA). The amplification was done at 95 °C for 1 min, followed by 35 cycles of amplification at 95 °C for 30 s, 51 °C for 30 s and 72 °C for 1 min, with a final extension step at 72 °C for 10 min. The amplification products were sequenced by Genewiz^®^ Society (Paris, France).

## Results

### Primer design

**Fig. 2:**
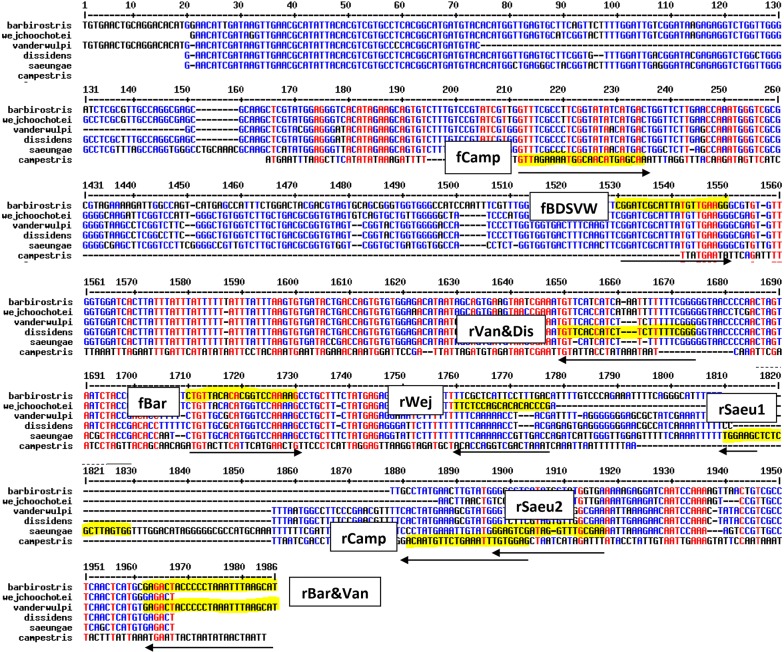
Alignment of ITS2 sequences of six species members of the Barbirostris Complex. The primer selection sites are highlighted in yellow and the corresponding primer names are written in white rectangles

**Fig. 3:**
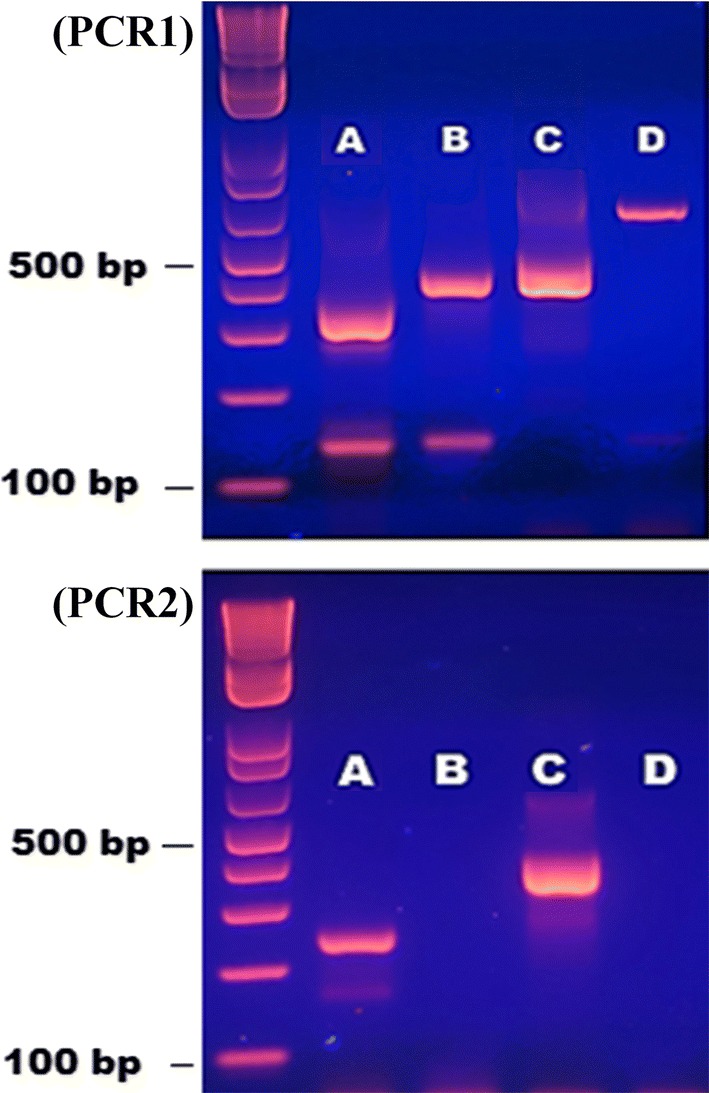
Multiplex PCR products from PCR1 (top gel) and PCR2 (bottom gel) of species of the Barbirostris Complex run on 2% agarose gel. For PCR 1 and 2, Lane A: *An. wejchoochotei*; Lane B: *An. dissidens*; Lane C: *An. saeungae*; Lane D: *An. campestris*. The fragment sizes of the DNA ladder are indicated in base pairs (bp)

Primer design was based on the ITS2 sequences of five out of six species available in GenBank (Table 2); the ITS2 sequence of *An. campestris* is not available. Primer names, sequences, size of the PCR products and respective melting temperatures (Tm) are presented in Table 3. Due to the close similarity of the ITS2 sequences of the six members, primer design was difficult with some species having similar individual size bands (bp), thus a dual PCR assay was developed in order to more reliably separate all species. Based on nucleotide alignment of the ITS2 region, nine primers were designed. A forward primer common to five species, except *An. campestris* (fBDSVW), then two species-specific forward primers (fBar, fCamp), and six reverse primers (rBar&Van, rVan&Dis, rCamp, rSaue1, rSaue2, rWej). *Anopheles barbirostris* is interrogated by two forward primers (fBDSVW and fBar) and one reverse primer (rBar&Van) providing 2 bands at 388 bp and 208 bp in PCR1 (Table 3). It was necessary to design a species-specific forward primer (fBar) for *An. barbirostris* in order to differentiate it from *An. vanderwulpi*, which shares the same two other primers (fBDSVW and rBar&Van). Nucleotide alignment of the amplified ITS2 region for the six species and the nine primer sites are shown in Fig. 2. The specificity of each primer was tested and the results presented in Fig. 3.

**Table 2:** Provenance of samples, GenBank accession numbers, and sources of the ITS2 sequences specific of 5 species of the Barbirostris Complex used for primer design (source: Table S1 in [2])

*Anopheles* species	Provenance of samples	GenBank ID	Reference
*An. barbirostris* (*n* = 3)	Chiang Mai, Thailand	AB971283.1	[2]
	Mae Hong Son, Thailand	EU812764.1	[23]
	South Kalimantan, Indonesia	EU812759.1	[23]
*An. dissidens* (*n* = 13)	Chiang Mai, Thailand	AB971284.1–AB971296.1	[2]
*An. saeungae* (*n* = 9)	Lampang, Thailand	AB971297.1–AB971305.1	[2]
	Trat, Thailand	EU812795.1	[23]
	West Sumatra, Indonesia	EU812791.1	[23]
*An. vanderwulpi* (*n* = 3)	West Sumatra, Indonesia	EU812766.1–EU812768.1	[23]
*An. wejchoochotei* (*n* = 8)	Chiang Mai, Thailand	AB971306.1–AB971311.1	[2]
	Sa Kaeo, Thailand	EU812808.1–EU812809.1	[23]

**Table 3:** Information on the nine primers designed for the dual multiplex PCR assay (PCR1, PCR2) for the identification of the six species of the Barbirostris Complex

Primer name	Specificity	Sequence (5′–3′)	CG %	Tm (°C)	Product size (bp)	PCR assay
fBDSVW	Common to 5 species	CGGATCGCATTATGTTGAAGG	47.6	47.3		1, 2
fBar	*An. barbirostris*	CTGTTACACACGGTCCAAAAG	47.6	47.3	208	1
fCamp	*An. campestris*	GTTAGAAAATGGCAACATGAGCAA	37.5	47.2		1
rBar&Van	*An. barbirostris*; *An. vanderwulpi*	ATGCTTAAATTTAGGGGGTAGTC	40.0	49.3	388; 401	1
rVan&Dis	*An. vanderwulpi*; *An. dissidens*	CCCGAAAAAGAAGATGGTGAACA	43.5	48.4	141; 141	1
rCamp	*An. campestris*	CTCCACAAATTTCAGAACATTGTCC	40.0	49.3	612	1
rSaeu1	*An. saeungae*	CACTAAGCGAGAGCTTCCA	52.6	58	294	2
rSaeu2	*An. saeungae*	TTCGCAAACCTATCGACTCC	50.0	60	378	2
rWej	*An. wejchoochotei*	GGGTGTGTGCTGGAGAAA	55.6	56	245	2

### Multiplex PCR

**Fig. 4:**
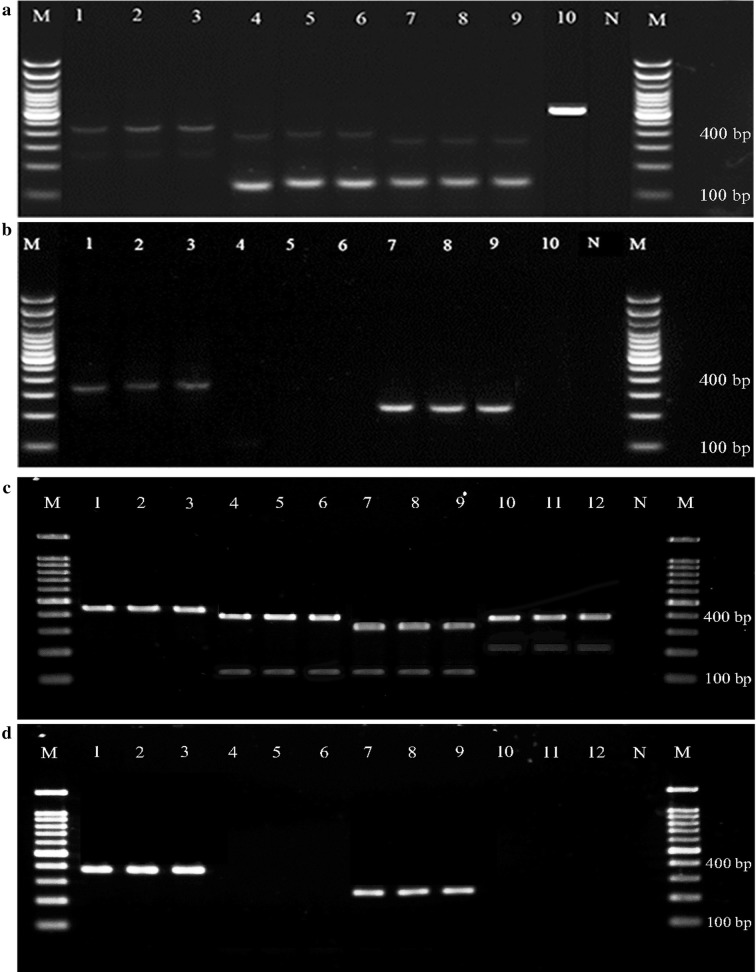
Multiplex PCR products from PCR1 (**a** and **c**) and PCR2 (**b** and **d**) of field specimens of the Barbirostris Complex run on 2% agarose gel. The fragment sizes of the DNA ladder (lane M) are indicated in base pairs (bp). **a**, **b** Lanes 1–3: *An. saeungae*; Lanes 4–6: *An. dissidens*; Lanes 7–9: *An. wejchoochotei*; Lane 10: *An. campestris*. **c**, **d** Lanes 1–3: *An. saeungae*; Lanes 4–6, *An. dissidens*; Lanes 7–9: *An. wejchoochotei*; Lanes 10–12: *An. barbirostris*

The three forward primers and the six reverse primers in the two PCR assays were able to differentiate simultaneously at least five members of the Barbirostris Complex. In PCR1, *An. barbirostris* and *An. dissidens* showed two bands: at 388 bp and 208 bp for *An. barbirostris* and at 410 bp and 141 bp for *An. dissidens* (Tables 3, 4, Figs. 3, 4). *Anopheles saeungae* presented two distinct bands, at 420 bp in PCR1 and another at 378 bp in PCR2; while *An. wejchoochotei* displayed three bands, at 335 bp and 141 bp in PCR1 and at 245 bp in PCR2 (Tables 3, 4, Figs. 3, 4). The purpose of developing a dual PCR, incorporating PCR2, was to confirm the differentiation between *An. saeungae* and *An. dissidens*, which have similar band sizes respectively at 420 bp and 410 bp in PCR1, while the second band at 141 bp present for *An. dissidens* may not always be visible. When all five species showed their respective band(s) in PCR1 (Table 4, Figs. 3, 4), there was no need for PCR2. Lastly, *An. campestris* was represented by a single band at 612 bp in PCR1. Primers have been designed for *An. vanderwulpi* but as this species is absent from Thailand, no specimen was tested. Most of the expected band sizes by species listed in Table 3 were confirmed in the AS-PCR (Table 4).

**Table 4:** Amplified fragment sizes in basepairs (bp) obtained for PCR1 and PCR 2 (when applicable) for five species of the Barbirostris Complex from Thailand

Species	PCR 1 (bp)	PCR 2 (bp)
*Anopheles barbirostris*	388; 208	–
*Anopheles campestris*	612	–
*Anopheles dissidens*	410; 141	–
*Anopheles saeungae*	420	378
*Anopheles wejchoochotei*	335; 141	245

### Sequencing of the ITS2 region

The complete ITS2 sequence length of each species ranges between 1500–1850 bp (Table 1). Within the 43 sequenced samples, three profiles were obtained from 31 specimens that matched the ITS2 sequences of *An. wejchoochotei* (*n* = 14), *An. saeungae* (*n* = 10) and *An. dissidens* (*n* = 7), respectively (Table 5). For the 12 additional sequences, a small part of the ITS2 sequence was amplified, which could not allow any definitive species identification.

**Table 5:** ITS2 sequences and corresponding GenBank accession numbers of 31 specimens belonging to three species of the Barbirostris Complex collected from 12 provinces in Thailand

Species	Province^a^	Province code	No. of isoline	Genbank ID
*An. wejchoochotei* (*n* = 14)	Chanthaburi (12)	CHN	2	MH796402, MH796403
	Kanchanaburi (5)	KAN	1	MH796405
	Prachuap Khiri Khan (8)	PRA	1	MH796406
	Ratchaburi (6)	RAT	1	MH796407
	Sisaket (9)	SRI	1	MH796408
	Surat Thani (14)	SUR	1	MH796409
	Tak (4)	TAK	1	MH796410
	Trat (13)	TRA	3	MH796411, MH796412, MH796413
	Ubon Ratchathani (10)	UBO	3	MH796414, MH796415, MH796416
*An. saeungae* (*n* = 10)	Chon Buri (11)	CBN	3	MH796417, MH796418, MH796419
	Phetchaburi (7)	PHT	1	MH796420
	Prachuap Khiri Khan (8)	PRA	2	MH796421, MH796422
	Ratchaburi (6)	RAT	1	MH796423
	Surat Thani (14)	SUR	2	MH796424, MH796425
	Tak (4)	TAK	1	MH796426
*An. dissidens* (*n* = 7)	Mae Hong Son (1)	MAE	1	MH796437
	Prachuap Khiri Khan (8)	PRA	2	MH796438, MH796439
	Surat Thani (14)	SUR	2	MH796440, MH796441
	Tak (4)	TAK	1	MH796442
	Ubon Ratchathani (10)	UBO	1	MH796443

^a^Number according to Fig. 1

### Distribution of the Barbirostris Complex species in Thailand

*Anopheles wejchoochotei* was identified in 54.6% of the specimens and in 19 of 23 sampled provinces indicating a wide distribution in Thailand (Fig. 1, Table 6). The second most common species was *An. saeungae* with 18.9% of the specimens collected in 15 provinces located throughout Thailand, excluding the southernmost region. *Anopheles dissidens* was found in 13 provinces, representing 24.3% of the collected specimens that were mainly located in the western and southern areas of Thailand. *Anopheles barbirostris*, with only 1.6% of the specimens identified, appeared confined to Chiang Mai Province, northern Thailand; while only one specimen (0.5%) of *An. campestris* was collected in Chanthaburi Province, east-central Thailand.

**Table 6:** Molecular identification of 185 field specimens of the Barbirostris Complex from 23 provinces of Thailand using the multiplex PCR assay developed in this study

Collection sites			Number of specimens				
Region	Province^a^	District	wej	saeu	diss	barb	camp
Northern	Mae Hong Son (1)	Khun Yuam	4		4		
	Chiang Mai (2)	Chiang Dao	3		3	3	
		Omkoi	5	4			
	Chiang Rai (3)	Mae Sai	4	3			
	Lampang (4)	Ko Kha		3			
Western	Tak (5)	Mae Ramat	4	1	1		
	Kanchanaburi (6)	Sai Yok	6				
	Ratchaburi (7)	Pak Tho	6	1			
	Phetchaburi (8)	Ta Yang	5	1			
	Prachuap Khiri Khan (9)	Huahin	6	4	5		
Northeastern	Buri Ram (10)	Lahan Sai	5	2			
	Surin (11)	Si Narong	5	2			
	Sisaket (12)	Phu Sing	6	1			
	Ubon Ratchathani (13)	Si Mueang Mai	8	1	1		
Eastern	Chon Buri (14)			3			
	Chanthaburi (15)	Makham	7		5		1
	Trat (16)	Borai	8	2	3		
Southern	Chumphon (17)	Pato	5	5			
	Ranong (18)	La-un			2		
	Surat Thani (19)	Panom	6	2	6		
	Phang Nga (20)	Thai Mueang			2		
	Songkhla (21)	Sadao	3		5		
	Yala (22)	Bannang Sata	3		5		
	Narathiwat (23)	Rueso	2		3		
Total (*n* = 185)			101	35	45	3	1
Frequency (%)			54.6	18.9	24.3	1.6	0.5

^a^Number according to Fig. 1

*Abbreviations*: wej: *An. wejchoochotei*; saeu: *An. saeungae*; diss: *An. dissidens*; barb: *An. barbirostris*; camp: *An. campestris*

## Discussion

Populations of *An. barbirostris* (*s.l*.) have been identified across a wide geographic range including India and Sri Lanka, throughout most of Southeast Asia, in particular Malaysia and Indonesia where it extends from Sumatra, Java, Bali in the west, to Kalimantan, Sulawesi, and throughout the Lesser Sunda Islands and Timor-Leste in the east [7]. Their role in transmission of both malaria and Brugian filariasis has been documented in Sulawesi, Flores and Timor [12, 13, 29–32], and mentioned as putative malaria vectors in Sri Lanka [33], possibly Bangladesh [34] and Thailand [10, 11, 17]. However, all of these reports on the natural vectorial role of *An. barbirostris* (*s.l*.) are based on unreliable morphological identifications and a period before the Barbirostris Complex was recognized in 2001 [35]. The complex now includes six formally named sibling species [1], and while almost identical (isomorphic) in adult morphology, their respective roles in malaria and filarial transmission differs drastically (Table 1). In Thailand, five formal species have been identified, while a sixth one, *An. barbirostris* species A3, has been reported from Kanchanaburi Province [25, 36]. Not formally characterized and unnamed, this species has a much smaller ITS2 sequence of 1070 bp [25]. Due to the lack of specimens, species A3 has not been included in this study, although primers have been designed to differentiate it from the other species of the complex. Considering the wide geographical distribution of the Barbirostris Complex and the involvement of some members in plasmodia and filarial transmission, it is crucial to better understand the full diversity of species that constitute the complex and which ones are vectors of public health importance so as to better target control efforts to maximize suppression of transmission and increase the likelihood of achieving malaria elimination [37]. Therefore, a reliable identification technique using molecular methods was an essential development to differentiate between species, especially those that occur in sympatry, that would allow further investigation on their specific bionomics, distribution and role in pathogen transmission.

For correct identification at the species level, molecular markers are used to separate sibling species, especially the rRNA ITS2 gene that is widely used to differentiate within many Asian *Anopheles* complexes [18–22]. The developed AS-PCR was able to differentiate all five species of the Barbirostris Complex present in Thailand. Primers to identify the species occurring in Indonesia, *An. vanderwulpi*, also found in sympatry with *An. barbirostris* [8], and *An. barbirostris* species A3 from Thailand have been designed and awaiting validation analysis of field samples when available. The application of this AS-PCR assay on 185 specimens collected throughout Thailand showed the wide distribution of *An. wejchoochotei*, previously presented as *An. campestris*-like species in a published distribution map [5]. This species is often found in sympatry with *An. saeungae* and *An. dissidens* based on collections from 13 and 11 provinces, respectively. *Anopheles barbirostris,* collected in one site only in Chiang Mai Province, was sympatric with both *An. wejchoochotei* and *An. dissidens*, while *An*. *saeungae* was the only species collected in Lampang (northern) and Chon Buri (eastern) Provinces. Only one specimen of *Anopheles campestris*, regarded an important vector of malaria and *Brugia malayi* filariasis [4], was collected in Chanthaburi Province, eastern Thailand.

## Conclusions

This study provides a simple, rapid, specific and efficient multiplex PCR assay for identifying the six described species members of the Barbirostris Complex. This assay has been validated on five species present in Thailand. Specimens of *An. vanderwulpi* from Indonesia and *An. barbirostris* species A3 from Thailand should now be tested using this AS-PCR in order to validate the primers. This multiplex PCR is a reliable identification tool for allowing a wide range of studies on the known species of the Barbirostris Complex. The assay also provides a tool with the possibility of recognition of new cryptic species populations throughout the broader geographical range of this complex.

## Data Availability

The datasets supporting the conclusions of this article are included within the article. The raw data used are available from the corresponding author upon reasonable request. Sequences are deposited in GenBank database under the accession numbers MH796402–MH796426 and MH796437–MH796443.
